# Patient and Provider Dilemmas of Type 2 Diabetes Self-Management: A Qualitative Study in Socioeconomically Disadvantaged Communities in Stockholm

**DOI:** 10.3390/ijerph15091810

**Published:** 2018-08-22

**Authors:** Juliet Aweko, Jeroen De Man, Pilvikki Absetz, Claes-Göran Östenson, Stefan Swartling Peterson, Helle Mölsted Alvesson, Meena Daivadanam

**Affiliations:** 1 Department of Public Health Sciences, Karolinska Institutet, SE-171 76 Stockholm, Sweden; helle.molsted-alvesson@ki.se (H.M.A.); meena.daivadanam@ikv.uu.se (M.D.); 2 Department of Public Health, Institute of Tropical Medicine, 43, 2000 Antwerp, Belgium; jdeman@itg.be; 3 Institute of Public Health and Clinical Nutrition, University of Eastern Finland, P.O. Box 111, FI-80101 Joensuu, Finland; ccsfinland@gmail.com; 4 Department of Molecular Medicine and Surgery, Karolinska University Hospital, SE-171 77 Stockholm, Sweden; claes-goran.ostenson@ki.se; 5 Department of Women’s and Children’s Health, International Maternal and Child Health, Uppsala University, 751 85 Uppsala, Sweden; stefan.peterson@kbh.uu.se; 6 Department of Food, Nutrition and Dietetics, Uppsala University, P.O. Box 560, 751 22 Uppsala, Sweden

**Keywords:** Self-management, type 2 diabetes, immigrants, health systems, chronic diseases, qualitative study, lifestyle change, thematic analysis, socioeconomically disadvantaged, Sweden

## Abstract

Studies comparing provider and patient views and experiences of self-management within primary healthcare are particularly scarce in disadvantaged settings. In this qualitative study, patient and provider perceptions of self-management were investigated in five socio-economically disadvantaged communities in Stockholm. Twelve individual interviews and four group interviews were conducted. Semi-structured interview guides included questions on perceptions of diabetes diagnosis, diabetes care services available at primary health care centers, patient and provider interactions, and self-management support. Data was analyzed using thematic analysis. Two overarching themes were identified: adopting and maintaining new routines through practical and appropriate lifestyle choices (patients), and balancing expectations and pre-conceptions of self-management (providers). The themes were characterized by inherent dilemmas representing confusions and conflicts that patients and providers experienced in their daily life or practice. Patients found it difficult to tailor information and lifestyle advice to fit their daily life. Healthcare providers recognized that patients needed support to change behavior, but saw themselves as inadequately equipped to deal with the different cultural and social aspects of self-management. This study highlights patient and provider dilemmas that influence the interaction and collaboration between patients and providers and hinder uptake of self-management advice.

## 1. Introduction

Self-management is the core strategy for people with Type 2 diabetes (T2D) to obtain glycemic control and prevent diabetes-associated complications [1,2]. Strategies recommended for successful self-management aim at empowering patients and include: diabetes self-management education [3,4,5,6] and support from health professionals, family and peers [7,8,9]. These are suggested to be effective in promoting patients’ ability to make decisions regarding the management of their illness [2]. Despite these strategies, self-management remains a challenge from both the patient and provider perspective. The responsibility of self-management is to a large degree the patient’s, who is expected to monitor and manage several aspects of the disease such as diet, physical activity, blood glucose, regular check-ups with health-care professionals [10] and adherence to treatment [3,11,12,13]. People with T2D are also expected to stay motivated [14,15], and develop skills including: decision-making, problem-solving, and translating knowledge into practical routines that enhance glycemic control [16]. This often involves adapting and maintaining a healthy lifestyle [17] which is a challenge for many of the people living with T2D [13,15,18]. One’s understanding of, perceptions, attitudes and beliefs regarding T2D [19,20] affect their decisions regarding self-management. Research has shown that people with T2D who perceive their disease as not serious are often less likely to engage in self-management than those who take their condition serious [19,21]. Other suggested factors hindering patients’ engagement in self-management include: limited support from health care professionals in terms of knowledge and guidance on making specific self-management plans, lack of understanding of treatment regimens and frustration associated with lack of glycemic control and continued disease progression despite adherence to recommendations [22]. These challenges are likely to be more pronounced among vulnerable populations particularly the socioeconomically disadvantaged and migrant groups due to possible cultural [23,24] and linguistic [25] barriers during clinical encounters. Healthcare providers on the other hand are expected to ensure that patients adhere to the recommended lifestyle advice which implies providing care that is tailored to the needs of the patient.

In Sweden, as in other countries, T2D is described as having a social gradient with people of low socioeconomic status [26] and migrants being disproportionately affected [27,28]. Low socioeconomic status is generally associated with poor health outcomes, poor health literacy and low utilization of healthcare services [26,29,30]. The proportion of migrants in Sweden is growing due to consistent increase in migration [31]. A large share of Swedish migrants are born outside Europe (mainly from the Middle East and Africa) and a majority live in socioeconomically disadvantaged suburbs of the country’s capital Stockholm [31]. The risk and prevalence estimates of T2D are reportedly higher in these suburbs than the county and national estimates (12–16% in adults 65 years and older compared to 5% and 6%, respectively) [32,33,34,35]. Whilst a great deal of research has been done in the medical management of diabetes, little effort has been directed towards understanding how people with diabetes perceive and experience self-management. Available studies have mainly focused on understanding patients’ attitudes, beliefs and perceptions regarding diabetes diagnosis, coping strategies [1,20,24,36,37] and social support [9] among people from specific ethnic groups, mostly from African and Middle Eastern backgrounds. Those studies highlight linguistic and cultural barriers in the interaction between patients and health professionals [20,24] and recommend culturally sensitive approaches and support for self-management. Studies comparing provider and patients views and experiences of self-management in primary healthcare settings are particularly scarce in socio-economically disadvantaged communities [38]. Available studies are mostly quantitative [39,40,41] and mainly focus on perceptions of T2D in the general population. Only a few have specifically studied perceptions of diabetes care among socioeconomically disadvantaged populations [40,41]. These studies suggest differences in patients’ and providers’ views of diabetes, which have documented consequences for diabetes self-management [40,41] that may be further exacerbated in vulnerable populations. This study sought to understand how self-management is perceived among persons with T2D born outside Europe and healthcare providers in socioeconomically disadvantaged communities in Stockholm.

## 2. Materials and Methods 

Data for this study were collected from July 2015 to October 2016, as part of the formative studies of a four-year collaborative research project (SMART2D) funded by EU Horizon 2020 [42].

### 2.1. Setting

The study was conducted in five socioeconomically disadvantaged communities together with their public primary healthcare centers located in three districts with a high proportion of immigrants (≥36%) in Stockholm County [43]. The population in the study setting comprises a mix of more than 100 nationalities, the majority originating from the Middle East (mostly Syria and Iraq) and Africa (mainly Somalia and Eritrea) [31], some newly arrived and others having lived in the areas for decades. Education levels vary among the residents in these communities, but income levels are low, unemployment levels are high and housing is generally poor compared to other parts of Stockholm County [26,30]. The means of the Care Need Index (CNI)—a measure of social deprivation—for deciles is also higher in these communities than in the affluent neighborhoods in the county [44]. Diabetes care is delivered in primary healthcare centers by teams of health professionals, mainly doctors and nurses, with referral to secondary and tertiary care, i.e., dieticians, podiatrists, ophthalmologists and endocrinologists where needed [45,46]. National guidelines for diabetes prevention and care exist and optimal diabetes care includes regular eye examinations, foot care, monitoring of glycemic control, blood pressure and cholesterol checks, all of which are essential in minimizing and preventing diabetes-related complications [47]. Registered patients with T2D receive subsidized care from a healthcare center of their choice with two annual follow-up visits [46] as in other parts of the county. One follow-up visit is with a doctor for structured individual tests including HbA1c, cholesterol and blood pressure and the other is with a diabetes nurse for lifestyle education [46]. 

### 2.2. Study Design and Approach

This study applied a qualitative design with individual and group interviews to explore patient and provider perceptions and experiences of T2D self-management. Three types of participants were involved: patients, healthcare providers and healthcare center managers. The selection of the patients study sample aimed to include participants with different nationalities, duration of diabetes, length of stay in Sweden, employment status, age and educational background, as a means to accommodate diversity in the study setting [48,49].

### 2.3. Participant Selection

By means of purposive sampling, patients were sequentially selected in two steps from a database of T2D-screened patients in the five healthcare centers. Selection of the study centers was based on their location in socioeconomically disadvantaged communities and their involvement in a screening program. Two of the healthcare centers had, at the time, an ongoing T2D screening program run by the 4D project, jointly managed by Karolinska Institutet and the Stockholm County Council [50].

Initially, we identified participants from these two healthcare centers who had been diagnosed with T2D in the last six months and had lived in Sweden for at least five years. The participants were identified from patient lists and each patient was contacted by a diabetes nurse and asked if they were interested in participating in the study. Interested participants were then scheduled for an interview by the research team. In a second step, after preliminary analysis, additional participants who had lived with a diabetes diagnosis for more than five years were included from the three other healthcare centers in order to obtain more variation in the perceptions of self-management in terms of period lived with diabetes.

Doctors and nurses specialized in T2D, who had daily or weekly contact with T2D patients, were purposively selected in order to obtain their view of self-management practiced by patients at the five health centers. In addition, four health center managers of the respective centers were interviewed to better understand the structure of diabetes care including the composition of providers’ and patients’ journeys from the community to the health center and referral to tertiary care. One of the managers was responsible for two of the healthcare centers and thereby presented views related to both centers.

In total, twelve patients, seven providers (including, doctors and nurses) and four managers were interviewed (see Table 1 and Table 2). The patient participants were born in seven different countries in four regions: Asia (Pakistan, Iran, Turkey and Syria), South American (Chile) and East Africa (Eritrea) and western African (Ivory Coast) while the health providers and managers were born in Sweden except three (one born in Africa, one in South America and the other in Middle East). The median length of stay in Sweden was 22 years and the level of education varied between primary school and university. Half of them were unemployed (see Table 1). The healthcare providers and managers had an average work experience of 10 years (see Table 2).

### 2.4. Data Collection

An interview guide with open-ended questions was developed to explore participants’ perceptions regarding four topic areas: (1) perceptions of diabetes diagnosis; (2) diabetes care, patient and provider interactions; (3) experiences of diabetes self-management; and (4) support for self-management. The initial interview guide was developed in English as a generic version for three field-site partners of the SMART2D project, informed by literature review on diabetes care [42]. The guide was further adapted to the Swedish context based on literature, practice and experience of investigators related to diabetes care in Sweden. The guide was pretested with three patients (not included in the study) to check for readability of the questions, suitability of the language and content. Minor revisions were made for purposes of comprehension (Appendix A). Additional questions and probes were added during the interviews in order to gain a deeper insight of participants’ perceptions of self-management.

The patients were free to choose the language they felt comfortable using during the interviews as well as the time and venue for the interview. The majority preferred to be interviewed in basic Swedish with the exception of one participant for whom an interpreter was used to translate from Swedish to Persian. The interviews were conducted by the first author (JA) together with a research assistant in the SMART2D project. The interviews took place in quiet spaces within libraries, participant homes, cafés and secluded spaces or rooms within the healthcare centers. Field notes were also taken during the interviews to aid understanding of the interview data and four member checks were conducted to seek clarification and affirm the meaning of responses [51]. The interviews lasted between 55 and 90 min. Based on preliminary analysis of the first nine interviews, three additional interviews were conducted in October 2016 to follow up on emerging concepts such as “using numbers to communicate diabetes”.

Providers were interviewed at the health centers in their working teams which comprised of groups of 2–3 participants per session. The managers were also interviewed in a group during a managers’ meeting at the county council composed of three participants. One of the managers, who was not present at the meeting, was individually interviewed. JA and a co-author (HMA) together moderated the interviews using a modified version of the interview guide previously used to interview participants with T2D (see Appendix A).

### 2.5. Data Analysis

All the interviews were recorded and transcribed verbatim in Swedish. The data were analyzed using Braun and Clarke’s thematic analysis [52,53] in three steps. Transcripts were entered into Nvivo software version 11 and an initial coding list based on preliminary analysis of the first three interviews was developed by JA and HMA. The codes were translated from Swedish to English and revised multiple times until a final list of codes was agreed upon and used to code the remainder of the interview data. Secondly, codes on self-management were compared and contrasted between the three categories of participants to identify themes and sub-themes that were assessed and refined by JA, HMA, and MD. Finally, responses from the patients and providers/managers were labeled as different types of dilemmas.

### 2.6. Ethical Approval and Participation Consent

The study was granted formal ethical approval by the Regional Ethics Review Board in Stockholm (Ref no. 2015/712-31/1). Permission to conduct the study was obtained from the heads of the participating primary healthcare centers. All participants gave their individual written informed consent prior to participation in the interviews.

## 3. Results

Two main themes were identified characterized by inherent dilemmas representing confusions and conflicts that patients and providers experience in their daily life or practice: adopting and maintaining new routines through practical and appropriate lifestyle choices from the patients’ perspective and balancing expectations and pre-conceptions of self-management from the providers’ perspective. Providers and managers shared some views on self-management and when this was the case, the views were presented together in the text as healthcare providers’ perspective. The dilemmas are informed by four sub-themes presented in Table 3.

### 3.1. Diabetes as Part of Aging and Family History

All of the patients in the study acknowledged that T2D is a part of the aging process dependent on their perceived genetic profile. The participants thought that it was normal to get T2D as one becomes older, as the majority had reportedly observed close relatives suffering from the disease. They also acknowledged that T2D was chronic but did not perceive it as fatal and reported that although one could have it for life, it was not as serious as having cancer or high blood pressure.

“… no, diabetes is sugar and a little sugar in the body is ok. Dad also had it, it is common to have diabetes.”

(Male with T2D)

Providers echoed that many of their patients did not perceive themselves as ill and did not take their condition seriously enough. As a result, they felt that their patients were not keen on changing their dietary and physical activity behavior as per their recommendations.

“… They do not know they are sick. They say that they have a little diabetes, a little sugar that the body gets used to …”

(Health provider)

### 3.2. Glucose Control through Numbers

Self-monitoring of blood glucose was a more common practice among younger patients than among older patients.

All patients reported that it was easy to interpret diabetes control through numbers. The “numbers” referred to are readings from a blood glucose test that participants conducted at home or at the healthcare center. Participants used these numbers to interpret their sugar levels as high or low, which also gave them a reason to take action. For example, many participants reported perceiving a high blood sugar value as a signal to immediately exercise in order to bring the numbers back to normal. The ability to change the numbers by walking or running also gave them a sense of control over their blood glucose.

“Today I took the blood sugar test. I had about 7.6 that is ok. I panic when I get more than about 8 or 9.”

(Female with T2D)

Concurrently, providers also acknowledged using numbers as an easy way to communicate and illustrate to the patients the seriousness of their illness. They also expressed that patients showed more concern and motivation to control their blood glucose when providers quoted numbers from patients’ blood glucose test readings.

“… If you tell the patient that their blood sugar is high, you see no reaction, but if you give the numbers, you get a different reaction. And if you say then that it has improved, and then justify it with the numbers, then they get motivated.”

(Health provider)

### 3.3. Tailoring of Information and Advice to Fit Daily Life

All patients received self-management advice from a healthcare provider during their visits to the primary healthcare center, and they were informed about the need to change diet and physical activity habits and to stop alcohol and tobacco use. However, they found it challenging to fit the diet and physical activity recommendations into their day-to-day life. Some participants described that they adopted specific practices that were easy to follow. These included: reducing their intake of sweet foods such as pastries, candy and sugar in tea; adding more fruit and vegetables to their daily meals; or performing simple exercises such as walking after meals. The older patients who had had T2D for a longer time only took medication to control their blood glucose and largely ignored the lifestyle recommendations. They felt that the diet and physical activity recommendations were impossible to perform since they had several concomitant conditions (including heart disease).

“I am on medication for heart disease and high blood pressure. Diabetes is just one of the conditions that I am being treated for. At the health center they tell me what to eat and that I should walk at least one hour but I don´t like it. I am always tired and I have back pain so I cannot do much.”

(Female with T2D)

The patients further pointed out that the lifestyle information they received from healthcare providers was not explicit as to what diet and physical activity practices were suitable for them or how to do them.

“… they told me, you must reduce your food, and exercise a lot … They just say you have to do that and that and that, but don’t tell you how. I need a plan with information on what to eat.”

(Male with T2D)

A predominant concern was the uncertainty surrounding which foods were suitable for consumption.

All healthcare providers had the expectation that patients should take responsibility for self-management. However, a majority of them were faced with a dilemma of deciding how much of the responsibility should be left to the patients. They tried multiple strategies for dealing with the patients. Some perceived their own role as giving patients instructions on what to do to manage their diabetes. Others mentioned that most of the patients do not follow the lifestyle advice recommended to them and attributed the above to the patients not taking their condition serious. A majority thought that self-management tasks were too difficult for the patients who were already dealing with several other socioeconomic concerns including; family responsibilities, unemployment and poor accommodation situations. They felt that it was their responsibility to support these patients.

“It is the patient’s responsibility, but a difficult one too. I often tell new patients that it is your responsibility but as a physician, you cannot just leave the responsibility to the patient. You cannot just say that it is his responsibility, you have to help them.”

(Healthcare provider)

The providers above mentioned that they were balancing roles in order to support the patients, i.e., they reported switching roles between “expert” and “facilitator” depending on the patient’s circumstances. A few providers took on the facilitator role to support mostly the elderly participants and the illiterate. They did this by learning about the cultural food habits of these patients and listening to different patients’ opinions about treatment alternatives. Such cultural experiences were reported useful in the provision of appropriate lifestyle advice.

“… what I have discovered is, I have patients that have different food habits. So first I have to learn the culture and what they eat, and then I have to be able to give advice. There are Somalis, so you have to be able to learn their food. For example, I did not know that they eat a banana every meal.”

(Healthcare provider)

Other healthcare providers found it difficult to adjust their roles, citing barriers such as a heavy workload and limited time for patients. Another challenge reported from the managers was the high staff turn-over in the health centers and lack of adequate resources to provide specialized care to the patients.

### 3.4. Frustrations Associated with Sustaining Change

All the patients acknowledged that managing T2D was a lifelong responsibility and felt that their lives were restricted. This was mainly attributed to the strict dietary practices they felt they had to follow. Most patients felt trapped by the disease and all its requirements. They expressed that they had no freedom to eat whatever they wanted—including their favorite foods—like they did before they were diagnosed and this had affected their social lives. Some patients expressed that they no longer wanted to go to social gatherings because it was frustrating for them to watch others eat everything while they had to be restrictive in what they ate.

“… It is frustrating when you are out at barbecues with friends. Then they eat more while you eat just a little. No, I want to eat normally. I want to eat like them …”

(Male with T2D)

The patients also expressed that sustaining dietary and physical activity routines was difficult as most of them had busy schedules, either working or taking care of their families, which they had to prioritize over their diabetes. These difficulties were mainly expressed by women. They further reported that the difficulties were felt more during festive seasons, holidays and trips abroad as they had limited food choices and found it difficult to maintain the new routines.

Other participants voiced feelings of frustration at not seeing substantial change in their blood glucose values even after persistently carrying out the new routines. In addition, participants who had lived longer with T2D (≥5 years) perceived self-monitoring of blood glucose as stressful and causing anxiety. For these participants, blood glucose was monitored only during medical consultations.

“… No, I no longer test my sugar at home, I used to get stressed and worried every time I tested my sugar. So I decided I will only do that when I go to the health center.”

(Male with T2D)

Healthcare providers in turn expressed concern about some of the participants and referred to them as “difficult patients”. These predominantly included those who not only had consistently high blood glucose values that they were unable to control even after several follow-up visits, but also others who were unreachable and lost to care because they travelled often or lived for longer periods in their home countries.

“… sometimes we call and call and do not reach the patient. Maybe they have moved or are abroad for about 10 months. I don’t know what they do in their home countries. Some come back but with high glucose values. Others don’t come, so we also give up.”

(Healthcare manager)

The providers felt the need to empower their patients to change behavior through education. However, they viewed diversity in culture pertaining to food, physical activity and treatment perceptions as a barrier to interaction. The patients had various cultural beliefs and perceptions of care that the providers found challenging to address.

“What is problematic then is that, the perception of food, treatment and physical activity is perhaps different from what we are used to.”

(Healthcare manger)

Additionally, it was reported that many persons in the study areas were mobile and often moved to better neighborhoods when their incomes improved or relocated to their home countries. This was experienced as a barrier to continuity of care.

### 3.5. Differing Views of Self-Management Support

For most patients, support for self-management was mainly expressed as a need for assistance with practical daily diet and physical activity tasks, mostly from close family members. Patients recognized family members (mainly spouses and children) as their core support in performing daily self-management tasks. These tasks included preparing meals for them based on lifestyle recommendations from their providers, accompanying them to clinic visits and encouraging them to exercise. Irrespective of which country they came from, men tended to rely more on support from family, particularly spouses, while women were more involved in deciding, and preparing their own meals.

“They try to help me with everything. My wife is trying to help me with the cooking and such. My wife watches what I eat. She makes a lot of salad, fish, chicken and little rice and no sugar.”

(Male with T2D)

In some instances, women received support and encouragement from older children for physical activity. Additionally, family members and relatives who were already managing T2D themselves were considered supportive in encouraging participants to change behavior. A few participants expressed frustration about family members who continuously instructed them on what to eat and avoid during meal times.

The patients also recognized that friends, work colleagues and peers could be helpful in motivating them to be physically active through conducting joint physical activities. However, there was an overall low expectation of help from this group due to lack of time and different work schedules, which did not allow them to meet regularly. Some men in particular were not keen about involving friends and peers in the management of their diabetes. They mentioned that, in their culture, illness was a private problem shared only with close family and healthcare providers. Others felt that no one else understood the experience of living with diabetes better than they themselves, leaving them with the responsibility of managing their own diabetes.

“… they are not with you every day to choose for you what you should or should not eat or what you should or should not do. You are alone with your diabetes … no one can help you except yourself …”

(Male with T2D)

In comparison, several women were engaged in some form of network involving activities for only women, e.g., water aerobics. They further expressed interest in peer group activities pertaining to physical activity exclusive to women as such activities gave them the opportunity to interact with fellow women.

“… A separate group is better because women have this thing, if it is a walk, men go fast but women are not as fast as men and during walking we can discuss and talk about some things … When women are alone, I do not know, but it’s better that way.”

(Female with T2D).

Healthcare providers recognized the value of family support during self-management and acknowledged that most of their patients were accompanied to consultations by family members often spouses and/or children. However, providers could not see the role of family members in the consultation rooms and in the facility care process. The presence of relatives in the room was often perceived as an inconvenience. The providers were uncertain about how to address relatives and at the same time maintain patient privacy. They also added that there was hardly any engagement with the patients beyond the patient-provider interactions during the short consultations and that very few patients participated in other clinic-led activities, such as outdoor walking activities.

## 4. Discussion

We identified two overarching themes characterized by inherent dilemmas in T2D self-management that patients and providers experienced in their daily life or practice respectively, which are important to consider while providing health care that meets the needs of population. These are discussed in terms of four main aspects that influence self-management listed below.

### 4.1. Diabetes Perceptions

Irrespective of country of birth, all patients in the present study perceived their disease as chronic but not fatal compared to other conditions such as cancer and hypertension. Similar perceptions of T2D have been well-documented [20,36,54,55,56]. According to these studies, some patients were found to not understand the severity of the disease [55,56] while others viewed the occurrence of their disease from a religious and cultural perspective as fated by a higher power [36,54]. In the present study, the perception that T2D was not fatal could have been influenced by the fact that most of the participants, particularly the elderly, had multiple conditions that they were managing and diabetes was only one of the many problems, thus was not given priority. In previous studies [36,54], patients of African and Middle Eastern descent who were found to perceive T2D as not serious attributed the disease to fate or supernatural factors such as God or Allah [36,54]. Such views have been shown to have consequences in patients’ uptake of lifestyle advice [57], often causing frustration for providers.

Provider perceptions in the present study reflected the frustrations inherent in their belief that patients do not understand the seriousness of the disease. Similar findings have been seen in previous studies [3,13]. Since patients did not perceive themselves as ill; providers felt that patients did not give due seriousness to their condition and hence did not take adequate measures to control their disease. In contrast to providers’ view, a possible reason for patients’ lack of seriousness could be attributed to unfamiliarity with the signs and symptoms of T2D and thus a tendency to normalize these symptoms. Normalizing signs and symptoms of disease can be due to inadequate acknowledgement of the seriousness of disease, leading to lack of urgency to change behavior [21,54,58].

### 4.2. Glycemic Control for T2D

Providers and patients found it easy to communicate diabetes control status using numbers from blood glucose test readings. Patients understood the current status of their glycemic control and what they needed to do to achieve glycemic control. The numbers gave patients a more concrete understanding of what action to take at which time point. Our findings are in support of Andersen and Whyte’s view that numerical values such as glucometer measurements are perceived as true indicators of health, and contribute to a formative process that motivates or provokes an individual to do something about their risk or condition [59].

Numeracy skills, i.e., “the ability to understand and use numbers in daily life”, are used by diabetes patients to assist with self-management tasks such as understanding glucose measurements, calculating medication doses (especially insulin) and interpreting dietary recommendations and nutrition labels [60]. High performance on a numeracy test developed and tested for diabetes has been found to have a significant correlation with higher diabetes knowledge and higher perceived self-efficacy [60,61] but the association between numeracy skills and glycemic control has not been consistently demonstrated [62,63,64,65]. In populations which face language barriers, numbers can become the single most important criterion for understanding disease status. From a medical viewpoint, reducing T2D to numbers without understanding what they mean could have adverse implications for care in the long term, since the numbers could denote short-term or long-term glycemic control depending on the test (e.g. fasting blood glucose and glycated hemoglobin, HbA1c).

However, from a medical viewpoint, reducing the disease (T2D) to numbers without understanding them could have adverse implications for care in the long term. Depending on the blood glucose tests used, the numbers could denote short-term glycemic control (random, fasting or post-prandial blood glucose) or long-term glycemic control (glycated hemoglobin, HbA1c). In Sweden, blood glucose control at the health center is mainly evaluated using HbA1c. However, patients often depend on point-of-care blood glucose tests using glucometers to self-monitor their diabetes. Short bursts of physical activity or dietary changes as a response to a high fasting or random blood sugar value as described by our participants, if interspersed with longer periods of sedentary or unhealthy lifestyle, could potentially result in fluctuations in blood glucose levels. Such fluctuations, occurring over a period of a few weeks to months, are referred to as “longer-term” glycemic variability, which is associated with a higher risk of microvascular complications, such as renal disease, independent of HbA1c values [66]. Similarly, unstructured exercise or dietary changes in response to high HbA1c levels may only lead to the erroneous perception of lower blood glucose without an actual reduction. A recent meta-analysis found that only structured physical activity lasting more than 150 min a week resulted in HbA1c reductions of 0.89% [67]. A significant and consistent reduction in HbA1c levels will most likely require sustained physical activity over a period of a few weeks. In patients using blood glucose test numbers as spontaneous triggers for physical activity and dietary changes, efforts are likely to be inconsistent. Moreover, a lack of regular physical activity and glycemic variability are both associated with fatigue, which could further undermine an individual’s ability to engage in self-management activities [68].

### 4.3. From New Routines to Sustainable Change

Patients found it difficult to implement self-management and struggled to adapt to the recommended lifestyle changes. Their main challenge was identifying suitable lifestyle practices and the practicalities of sustaining new routines, especially in relation to social occasions and holidays. A major dilemma was uncertainty about what foods were appropriate for consumption and in what proportions. This dilemma was also acknowledged by the providers, although they did not seem to consider this as a priority, being more concerned with providing clinical treatment. Moreover, providers perceived their responsibility to be restricted to providing instructions on self-management. These findings are consistent with those of another study in a similar setting in Sweden [20,54], where participants with a Somali background reportedly found it difficult to follow dietary advice and perceived it as culturally inappropriate.

In the present study as in previous studies [13,21,22,55], adopting and maintaining new routines, particularly those related to diet and physical activity, was a challenge for all participants with T2D. The dilemma that patients expressed relating to uncertainty of what foods are appropriate for consumption is also recognized as a challenge among participants practicing self-management in another study [13]. Booth et al. [13] found that, despite attending several lifestyle education sessions, participants still express uncertainty about which foods are suitable or unsuitable for consumption. As a result, patients try to find what works for them by selecting practices that are easy to perform, and these are most times already embedded in their personal daily routines [3]. Possible explanations could be that the patients do not receive adequate information regarding self-management or the lifestyle advice given by providers is too broad and does not take into account the different cultural and social needs of the targeted patients, thus making it difficult for them to follow the lifestyle advice recommended. 

### 4.4. Self-Management Support

The present study, along with earlier studies [3,9,69], reveals that the engagement of close family in self-management is perceived by patients to be very important since it assists them with daily self-management tasks. Need for support in daily self-management tasks, specifically deciding what food to prepare and how to prepare it was mostly reported by men than women. Similar views were voiced by the providers, although they expressed a dilemma in dealing with patients together with their relatives in the same consultation room and at the same time maintaining privacy. Gender differences in perceptions of family support have also been documented in other studies [3,70,71]. Men often depend on their spouses for support in self-management tasks while women tend to utilize their extensive network for support in stressful situations [3,71]. Such differences could be influenced by the traditional or cultural backgrounds of the participants.

Support from close family members could enhance autonomous self-management among participants as a result of collective performance of self-management tasks, such as attending consultation visits together and offering emotional encouragement [14,69]. Research suggests that support for self-management education and awareness-raising is a means to achieve behavior change [17]. The focus of self-management education has shifted to building the “how” and the necessary skills to go with it, together with empowering and motivating individuals [17]. Some providers in the present study took the initiative to balance their expert and facilitator roles during consultations, i.e., listening to participants’ opinions about their treatment and giving patients advice according to their needs. This is a shift towards patient empowerment and a patient-centered approach to chronic disease management, which advocates collaboration between providers and patients in order to help patients achieve their own health goals [5]. However, providers’ efforts are often inadequate as they are not sufficiently equipped to handle the different cultural and social needs of their patients. Cultural incompetence among providers has been highlighted as a challenge and as a result, general practitioners reportedly ignore cultural differences during meetings with immigrant patients because they were uncertain of how to deal with the different cultural needs of the patients [24]. Moreover, providers were seen to face a dilemma with respect to patients’ role in self-management; they expect the patient to take lead in the management of their diseases, but also think patients are incapable of managing their disease and thus they have to intervene [69].

Education level has previously been demonstrated to have more influence on illness perceptions and self-management than income [72]. In the present study, however, no conclusions could be drawn on the effect of education. Despite the variation in the education level among these participants, no major differences were noted in the perceptions or experiences of diabetes self-management. It could also be that, irrespective of level of education, the participants live in the same neighborhood and share similar problems and limited resources. Thus, they try to find a common ground to cope with the limited resources [73].

### 4.5. Methodological Considerations

Participant sampling is one of the challenges in the design and implementation of interventions, particularly in communities that are diverse in terms of nationality, ethnicity, and length of stay in the country, age, language and educational level. With the increase in diversity in Sweden, there is a need for the health care system and research projects to develop interdisciplinary and intercultural strategies that include all groups of people irrespective of their ethnicity. Interculturalism is one such approach that focuses on the common characteristics of the population and less on the differences in ethnicity, culture, sex, age, language, religion, etc., and suggests reciprocity and accommodation as means to address diversity [48,49]. A strength of this study is the consideration of the common characteristics of living in the same geographical location and being an immigrant among the population and using the above characteristics as a basis for selection of the patient sample. In that way, the views and experiences of different groups of people within the population are represented in the sample selected. For the same reason, the findings of this study are transferrable to other socioeconomically disadvantaged settings in high income countries with a high proportion of immigrants.

Another strength of the study is the multidisciplinary composition of the study team. It included doctors, a nutritionist, a medical anthropologist, a behavioral scientist and an endocrinologist, all with diverse public health skills and experience, which brought different perspectives to the study. The study sample was purposively selected in order to understand the perceptions of type 2 diabetes self-management from the perspectives of patients, providers and managers. Participants with T2D included only those with access to the health facility for care and it is likely that hard-to-reach groups within this population were not included. Our study sample provided sufficient information power to answer our research questions based on Malterud and coworkers’ description of information power [74]. The questions were specific and focused on understanding self-management and yielded robust data. In addition, triangulation of data sources and data collection methods made it possible to validate the core findings.

## 5. Conclusions

This study highlights dilemmas representing struggles and confusions that patients experience while adjusting to lifestyle recommendations and providers’ care practice. As patients struggled to adapt lifestyle recommendations to their everyday life, healthcare providers, sought to support the patients in this struggle, mainly through education, but felt that they were not adequately equipped to deal with the different cultural and social aspects of self-management. Understanding these dilemmas is important in diabetes care as they influence the interaction and collaboration between patients and providers and in turn impact the uptake and utilization of self-management advice and services by patients. Thus, they should be considered when designing self-management interventions in primary health care centers particularly in socioeconomically disadvantaged settings. Additionally, intercultural approaches should be further studied in dealing with diversity in the selection of study participants and implementing self-management interventions in socioeconomically disadvantaged settings with a high proportion of immigrants.

## Figures and Tables

**Table 1:** Characteristics of participants with type 2 diabetes.

Characteristics	Women	Men
Number of participants	6	6
Duration of diabetes		
<5	3	2
≥5	3	4
Length of stay in Sweden (in years)		
5–15	3	1
>15	3	5
Employment status		
Employed	3	3
Unemployed	3	3
Education level		
Primary school	2	2
High school	2	2
University	2	2
Age group		
35–59	2	4
>60	4	2

**Table 2:** Characteristics of healthcare providers.

Characteristics	Women	Men
**Number of participants**	8	2
**Profession**		
Doctors	1	1
Nurses	5	-
Healthcare managers	3	1
**Years of experience**		
<5	1	-
6–10	2	1
>10	5	1

**Table 3:** Results from participant interviews.

Themes: Dilemmas	Sub-Themes	Categories
Patient dilemma: Adopting and maintaining new routines through practical and appropriate lifestyle choices	Diabetes as part of aging and family history	Not as serious as conditions like hypertension and cancer (patient) Patients do not perceive diabetes as serious (provider)
	Glucose control through numbers	Interpreting diabetes control through numbers (patient) Easy to communicate numbers (provider)
Provider dilemma: Balancing expectations and pre-conceptions of self-management	Tailoring of information and advice to fit daily life	Insufficient information (patient) Adopting selective recommendations (patient) Ignoring impossible recommendations (patient) Cultural differences as a barrier to interaction (provider) Balancing opposing roles of self-management responsibility (provider)
	Frustrations associated with sustaining change	Realizing chronicity (patient) Stress and anxiety of monitoring glucose levels (patient) Belief in education to promote behavior change (provider) Coping with “difficult” patients (provider)
	Differing views of self-management support	Expecting support from family (patient) Low expectation of support from friends and peers Balancing between family support and patient privacy (provider)

## Appendix A

Semi-structured interview Guide for people with type 2 diabetes

Theme 1: Perceptions of type 2 diabetes diagnosis

1. Please describe the period just before you got the T2D diagnosis: how did you feel?

2. Tell us about the consultation when you received your diagnosis?

3. After you had been diagnosed, how did you react?

4. If you were to compare the reactions you got when you received diabetes diagnosis do you think that they would be the same as if you got a diagnosis of another chronic disease?

5. If you were to compare the reactions you got when you received the diabetes diagnosis with the reactions you have noticed that other people with diabetes got, do you think that these reactions are similar? What about people with high blood pressure—what reactions do they get? What about people with other chronic diseases?

Theme 2: Diabetes care, patient and provider interactions

6. Can you describe your contact with the healthcare center?

7. During your visits to the health center what do you talk about?

8. Please describe the information you receive during consultations at the healthcare center

9. What changes have you made in relation to your diet, smoking, physical activity after your diagnosis?

Theme 3: Experiences of diabetes self-management

10. Can you describe what you do on a daily basis to manage your condition?

11. Please describe how you feel and what you do to keep your blood sugar stable on a good day?

Theme 4: Support for self-management

12. If you think about your everyday life with diabetes—describe the support you have received?

13. What challenges have you faced during the management of your condition?

Semi-structured interview Guide for healthcare professionals and health managers

Theme 1: Diabetes care, patient and provider interactions

1. How is diabetes care organized at this healthcare center?

2. Describe the typical patient seeking diabetes care here?

3. How do patients react when diagnosed with T2D?

4. Please describe the type of treatment programs you offer newly diagnosed patients?

5. How do patients react to becoming diagnosed with diabetes

6. How do you describe self-management to the patients? How do they understand it?

Theme 2: Caregiver’s experiences and support for self-management

1. Do you offer other types of support to the T2D patients?

2. What challenges do you encounter in the treatment of patients with diabetes as compared to patients with other lifestyle-related diseases?

3. How do you view the need for diabetes prevention and primary health care treatment in areas with a large proportion of migrants in relation to areas where most people are born in Sweden?

4. Perceptions of disease causes can vary between different cultures and it can be difficult for the doctor and the patient to understand each other—Do you think this is a problem in diabetes care here?

5. To what extent do you think that proper care and support are offered to diabetes patients?

6. If a patient has trouble following treatment and recommendations, how is that managed?
